# Association between monocyte lymphocyte ratio and abdominal aortic calcification in US adults: A cross-sectional study

**DOI:** 10.1016/j.clinsp.2023.100232

**Published:** 2023-06-24

**Authors:** Peiyuan Zuo, Ranran Xu, Liya Hu, Wei Hu, Song Tong

**Affiliations:** a Department of Geriatrics, Tongji Hospital, Tongji Medical College, Huazhong University of Science and Technology, Wuhan, China; b Department of Pediatric, Tongji Hospital, Tongji Medical College, Huazhong University of Science and Technology, Wuhan, China; c Department of Thoracic Surgery, Union Hospital Tongji Medical College Huazhong University of Science and Technology, China

**Keywords:** Monocyte lymphocyte ratio, Abdominal aortic calcification, Vascular calcification, Cross-sectional study

## Abstract

**Background:**

This study aimed to evaluate the association between Monocyte Lymphocyte Ratio (MLR) and Abdominal Aortic Calcification (AAC) in adults over 40 years of age in the United States.

**Methods:**

Data were collected from the 2013–2014 National Health and Nutrition Examination Survey (NHANES). AAC was quantified by the Kauppila score system based on dual-energy X-Ray absorptiometry. Severe AAC was defined as a total AAC score > 6. The lymphocyte count and monocyte count can be directly obtained from laboratory data files. Multivariable logistic regression models were used to determine the association between MLR and the AAC score and severe AAC.

**Results:**

A total of 3,045 participants were included in the present study. After adjusting for multiple covariates, MLR was positively associated with higher AAC score (β = 0.21, 95% CI 0.07, 0.34, p = 0.0032) and the odds of severe AAC increased by 14% per 0.1 unit increase in the MLR (OR = 1.14, 95% CI 1.00, 1.31, p = 0.0541). The Odds Ratio (OR) (95% CI) of severe AAC for participants in MLR tertile 3 was 1.88 (1.02, 3.47) compared with those in tertile 1 (p for trend = 0.0341). Subgroup analyses showed that a stronger association was detected in the elderly compared with non-elderly (p for interaction = 0.0346) and diabetes compared with non-diabetes (borderline significant p for interaction = 0.0578).

**Conclusion:**

In adults in the United States, MLR was associated with higher AAC scores and a higher probability of severe AAC. MLR may become a promising tool to predict the risk of AAC.

## Highlights

• Elevated level of MLR associated with higher AAC score.
• Each 0.1 unit of increased MLR was associated with a 14% increased risk of severe AAC.
• This positive relationship between MLR and AAC score was stronger in the elderly and with diabetes.

## Introduction

Vascular Calcification (VC) pathology is characterized by ectopic calcification of the vascular wall of a muscular artery or elastic artery. Several studies have shown higher chronic kidney disease, Type 2 Diabetes (T2D), atherosclerosis, and cardiovascular morbidity and mortality for patients with vascular calcification.^1^, ^2^, ^3^ Currently there is no satisfactory treatment for VC. Intravascular lithotripsy may provide a new option for lesion preparation for severely calcified plaque in the coronary arteries and peripheral blood vessels.^4^ However, the cost-effectiveness of such technology will need to be considered. Therefore, it is of great significance to find the risk factors and preventive measures of VC.^5^

The common site of VC is Abdominal Aortic Calcification (AAC). AAC grade is an important predictor for cardiovascular disease mortality, which can be noninvasively quantified in clinical practice.^6^^,^^7^ Using lateral radiographs of the lumbar region can quantitatively evaluate the degree of calcification. Multiple studies suggest that age, smoking, obesity, dyslipidemia, and hypertension are possible risk factors for the development of AAC.^8^, ^9^, ^10^, ^11^, ^12^

Monocyte count is strongly associated with a higher risk of cardiovascular events and mortality.^13^ In addition, Monocyte Lymphocyte Ratio (MLR), a robust inflammatory biomarker, could help predict the risk of cardiovascular disease and assess the severity of coronary artery disease.^14^, ^15^, ^16^ The relationship between monocyte lymphocyte ratio and AAC has not been reported before. Therefore, using the 2013‒2014 National Health and Nutrition Examination Survey (NHANES) cohort, the authors assessed the association between MLR and AAC. The authors hypothesized that the monocyte-to-lymphocyte ratio was associated with an increased incidence of AAC.

## Methods

### Study population

This study from the National Health and Nutrition Examination Survey (NHANES) was approved by the National Center for Health Statistics (NCHS) Ethics Review Board. NHANES, is a national cross-sectional study designed to assess the health and nutrition status of adults and children in the United States. All participants underwent comprehensive measurements and standardized interview questionnaires, such as demographics, health-related questions, and laboratory examinations. Details of the NHANES are publicly available on the CDC website (https://www.cdc.gov/nchs/ nhanes/index.htm).

**Fig. 1:**
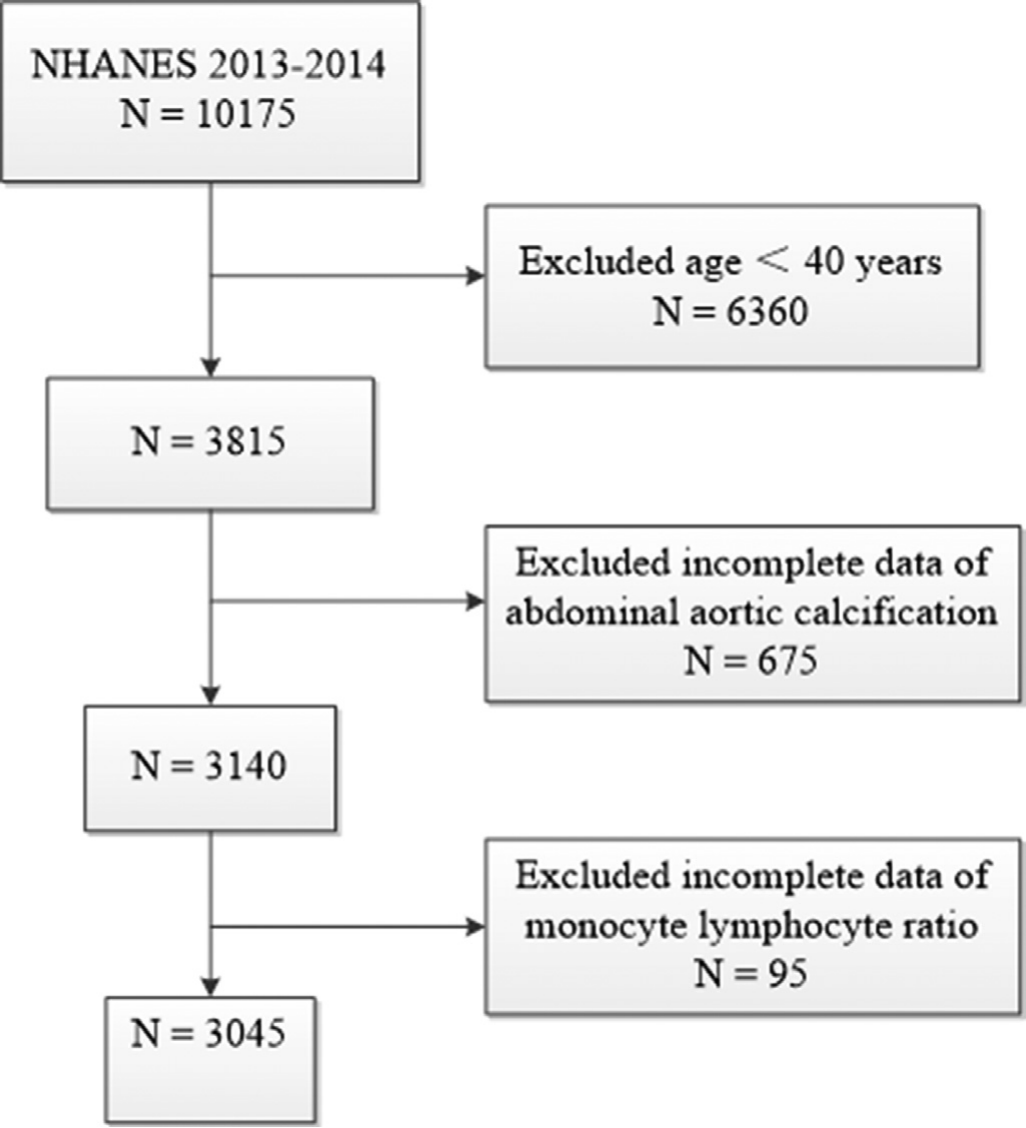
Flowchart of participant selection.

The authors used data from 2013 to 2014 NHANES, because this cycle includes data on MLR and ACC scores. A total of 10175 samples were involved in the interviews. The authors first excluded participants younger than 40 years for those individuals who did not undergo Dual-energy X-Ray Absorptiometry (DXA) scans during 2013‒2014 from the NHANES. The authors further excluded 675 participants missing data of AAC score and 95 participants missing data of MLR. Finally, a total of 3045 participants were enrolled in this study. The flow chart of the inclusion and exclusion criteria is described in Fig. 1.

### Exposure and outcomes

MLR, the monocyte count/lymphocyte count was designed as exposure variable. Both these data can be directly obtained on the CDC website from laboratory data files. The Beckman Coulter DxH 800 instrument in the NHANES Mobile Examination Center (MEC) produces a complete blood count on blood specimens and provides a distribution of blood cells for all participants.

The outcome variables were AAC score and severe AAC. AAC can be accurately recognized with Dual-energy X-Ray Absorptiometry (DXA), which can detect AAC with reasonably good sensitivity and specificity.^17^ AAC scoring with a range from 0 to 24 according to the Kauppila scoring method was used for the abdominal aortic calcification evaluation.^18^ The anterior and posterior aortic walls are divided into four segments, corresponding to the areas in front of the lumbar vertebrae L1‒L4. Within each of these 8 segments, a range from 0 to 3 scores were obtained according to the degree of aortic calcification. Severe AAC was designed as another outcome variable as well. As in previous studies, it was defined as a total AAC score >6, which represented significant aortic calcification lesions.^7^^,^^19^

### Covariates

The authors collected potential covariates that might confound the association between MLR and ACC score. Covariates included gender (male or female), age, race (Mexican American, Other Hispanic, Non-Hispanic White, Non-Hispanic Black or Other Race), an education level (Less than high school, High school or GED or Above high school), Smoker (Participants who had at least 100 cigarettes in life were considered to be smokers), Drinker (participants who had at least 12 alcohol drinks per year were considered to be drinker), Body Mass Index (BMI), Alanine Aminotransferase (ALT), Aspartate Aminotransferase (AST), Systolic Blood Pressure (SBP), Diastolic Blood Pressure (DBP), serum creatinine, Urea nitrogen, serum uric acid, hemoglobin A1c, total cholesterol, Folate and status of diabetes (Doctor or other health professional told informed you that youhave diabetes), hypertension (Doctor or other health professional informed you that you have high blood pressure), and kidney disease (Doctor or other health professional told informed you that you have weak/failing kidneys).

### Statistical analysis

Baseline characteristics of all patients according to stratification by monocyte lymphocyte ratio level were expressed as a proportion for categorical variables, mean ± SD or median, and interquartile for continuous variables. The differences between groups were analyzed using the chi-squared test for categorical variables, the one-way ANOVA for normally distributed continuous variables, and the Kruskale-Wallis test for skewed continuous variables.

Multivariate logistic regression models were used to explore the independent relationship between MLR and ACC (including AAC score and severe AAC) in three different models. In the adjusted regression model 1, age, gender, education level, and race were included. In the adjusted regression model 2, age, gender, education level, race, diabetes, hemoglobin A1c, BMI, AST, ALT, urea nitrogen, drinker, SBP, DBP, kidney disease, total cholesterol, smoker, uric acid, hypertension, and serum creatinine were included. The authors performed tests for linear trends by entering the median value of each category of MLR as a continuous variable in the models. Subgroup analysis stratified by gender, age, BMI (normal weight, overweight, and obese), diabetes, hypertension, and kidney disease was also performed using stratified multivariate regression analysis. All P values were calculated using two-tailed tests of statistical significance with a type I error rate of 5%. All statistical analyses were performed using Empower(R) (www.empowerstats.com, X&Y solutions, Inc., Boston, MA) and R (http://www.R-project.org).

## Results

The demographic characteristics of included participants stratified by MLR were shown in Table 1. A total of 3045 participants with a mean age of 58.6 ± 12 years old were included, of whom 48.34% were male. The mean MLR was 0.3 ± 0.14, and the ranges of MLR tertiles 1–3 were 0.07‒0.23, 0.24‒0.32, and 0.32‒1.36 respectively. Among different quartiles of MLR, significant differences were observed in age, gender, smoker, drinker, BMI, SBP, DBP, hypertension, kidney disease, alanine aminotransferase, aspartate aminotransferase, serum creatinine, Urea nitrogen, serum uric acid, Hemoglobin A1c, total cholesterol and Folate, AAC score, and the prevalence of severe AAC. Both the AAC score and the prevalence of severe AAC increase with the higher MLR tertiles. The mean AAC score was 1.63±3.5 overall, which was respectively 1.04 ± 2.47, 1.47 ± 3.27 and 2.34 ± 4.31 for MLR tertiles 1‒3 (p < 0.05). The prevalence of overall severe AAC score was 9.01%, which was respectively 4.33%, 8.14%, and 14.12% for MLR tertiles 1‒3 (p < 0.05).

**Table 1:** Baseline characteristics of participants according to Monocyte Lymphocyte ratio (MLR) level.

	Overall	Tertile 1 (0.07‒0.23)	Tertile 2 (0.24‒0.32)	Tertile 3 (0.32‒1.36)	p-value
		n = 969	n = 1056	n = 1020	
**Age (yr)**	58.60 ± 12.00	55.67 ± 10.60	57.69 ± 11.66	62.38 ± 12.60	< 0.001
**Men, n (%)**	1518 (48.34%)	326 (33.64%)	521 (49.34%)	621 (60.88%)	< 0.001
**Race, n (%)**	3				< 0.001
Mexican American	412 (13.12%)	147 (15.17%)	148 (14.02%)	107 (10.49%)	
Other Hispanic	298 (9.49%)	110 (11.35%)	114 (10.80%)	65 (6.37%)	
Non-Hispanic White	1375 (43.79%)	307 (31.68%)	464 (43.94%)	576 (56.47%)	
Non-Hispanic Black	620 (19.75%)	220 (22.70%)	180 (17.05%)	186 (18.24%)	
Other Race	435 (13.85%)	185 (19.09%)	150 (14.20%)	86 (8.43%)	
**Education level, n (%)**					0.352
Less than high school	726 (23.14%)	238 (24.56%)	242 (22.94%)	220 (21.59%)	
High school or GED	704 (22.43%)	219 (22.60%)	248 (23.51%)	220 (21.59%)	
Above high school	1708 (54.43%)	512 (52.84%)	565 (53.55%)	579 (56.82%)	
**Smoker, n (%)**	1405 (46.27%)	395 (40.76%)	486 (46.07%)	524 (51.42%)	< 0.001
**Drinker, n (%)**	2041 (71.29%)	573 (63.31%)	724 (73.50%)	744 (76.46%)	< 0.001
**BMI**	28.45 ± 5.58	28.64 ± 5.78	28.65 ± 5.37	28.08 ± 5.58	0.028
**Diabetes, n (%)**	506 (17.32%)	156 (16.76%)	189 (18.68%)	161 (16.43%)	0.359
**Hypertension, n (%)**	1441 (47.37%)	426 (43.96%)	485 (46.02%)	530 (52.01%)	< 0.001
**Kidney disease, n (%)**	117 (3.83%)	27 (2.79%)	35 (3.32%)	55 (5.40%)	0.006
**ALT (U/L)**	24.68 ± 18.34	23.08 ± 12.12	25.84 ± 24.12	25.02 ± 16.24	0.003
**AST (U/L)**	25.53 ± 14.04	24.09 ± 9.40	26.04 ± 17.41	26.32 ± 13.76	< 0.001
**SBP (mmHg)**	127.72 ± 19.12	125.94 ± 18.23	126.98 ± 18.77	130.18 ± 19.96	< 0.001
**DBP (mmHg)**	71.03 ± 13.30	71.70 ± 11.52	71.26 ± 12.82	70.14 ± 15.09	0.027
**Serum creatinine (µmoL/L)**	83.36 ± 46.18	75.97 ± 29.51	81.61 ± 31.21	92.23 ± 66.47	< 0.001
**Urea nitrogen (mmoL/L)**	5.11 ± 2.25	4.73 ± 1.75	5.03 ± 2.07	5.57 ± 2.73	< 0.001
**Serum uric acid (µmoL/L)**	324.24 ± 82.75	312.71 ± 75.93	324.43 ± 82.58	334.67 ± 87.60	< 0.001
**Hemoglobin A1c (%)**	5.93 ± 1.18	5.93 ± 1.16	6.01 ± 1.37	5.84 ± 0.96	0.003
**Total cholesterol (mmoL/L)**	5.04 ± 1.13	5.19 ± 1.17	5.08 ± 1.05	4.85 ± 1.10	< 0.001
**Folate (nmoL/L)**	1280.51 ± 592.04	1220.29 ± 568.62	1266.50 ± 554.01	1353.59 ± 644.01	< 0.001
**AAC score**	1.63 ± 3.50	1.04 ± 2.47	1.47 ± 3.27	2.34 ± 4.31	< 0.001
**Severe AAC, n (%)**	272(9.01%)	42 (4.33%)	86 (8.14%)	144 (14.12%)	< 0.001

Data are mean ± SD, median (interquartile range), or percentage.

BMI, Body Mass Index, SBP, Systolic Blood Pressure, DBP, Diastolic Blood Pressure, ALT, Alanine Aminotransferase, AST, Aspartate Aminotransferase, AAC, Abdominal Aortic Calcification.

Table 2 shows the univariate and multivariate model of β and 95% Confidence Intervals (CIs) for the ACC score determined by MLR. In the unadjusted model, no covariates were adjusted. Model I was adjusted for age, gender, education level, and race. Both the unadjusted model and model I showed a positive relationship between MLR×10 and AAC score (β = 0.48, 95% CI 0.39, 0.57, p < 0.0001 for an unadjusted model; β = 0.24, 95% CI 0.15, 0.33, p < 0. 0001 for model I). Model II was adjusted for age, gender, education level, race, diabetes, hemoglobin A1c, BMI, AST, ALT, urea nitrogen, drinker, SBP, DBP, kidney disease, total cholesterol, smoker, uric acid, hypertension, and serum creatinine. The positive association was still stable in the fully adjusted model (β = 0.21, 95% CI 0.07, 0.34, p = 0.0032), indicating that each 0.1 unit of increased MLR was associated with 0.21 increased unit of AAC score. The authors also converted MLR from a continuous variable to a categorical variable (tertiles). AAC score increased with the higher MLR tertiles group (p for trend < 0.0001 in the unadjusted model, p for trend = 0.021 in model I, p for trend = 0.0062 in model II). In a fully adjusted model, the AAC score of the highest MLR tertile (tertile 3) was 0.64 units higher compared with the lowest tertile (β = 0.64, 95% CI 0.18, 1.1, p = 0.0066).

**Table 2:** Multivariate logistic regression models of AAC score with MLR.

	Unadjusted β (95% CI)	Model I β (95% CI)	Model II β (95% CI)
	p-value	p-value	p-value
**MLR×10**	0.48 (0.39, 0.57) < 0.0001	0.24 (0.15, 0.33) < 0.0001	0.21 (0.07, 0.34) 0.0032
**Categories**			
Tertile 1	Reference	Reference	Reference
Tertile 2	0.43 (0.13, 0.73) 0.0048	0.16 (-0.13, 0.44) 0.2757	0.23 (-0.21, 0.66) 0.3111
Tertile 3	1.30 (1.00, 1.60) <0.0001	0.47 (0.17, 0.77) 0.0022	0.64 (0.18, 1.10) 0.0066
p for trend	<0.0001	0.0021	0.0062

The model I adjust for age, gender, education level, and race; Model II adjusts for: age, gender, education level, race, diabetes, hemoglobin A1c, BMI, AST, ALT, urea nitrogen, drinker, SBP, DBP, kidney disease, total cholesterol, smoker, uric acid, hypertension, and serum creatinine.

Table 3 shows the regression models of severe AAC with MLR. The risk of severe AAC increased as MLR increased. In the fully adjusted model, the authors adjusted age, gender, education level, race, diabetes, hemoglobin A1c, BMI, AST, ALT, urea nitrogen, drinker, SBP, DBP, kidney disease, total cholesterol, smoker, uric acid, hypertension, and serum creatinine. The result indicated that each 0.1 unit of increased MLR was associated with a 14% increased risk of severe AAC (95% CI 1.00, 1.31, p = 0.0541). Meanwhile, the risk of severe AAC increased with the higher MLR tertiles (unadjusted model, OR = 3.63, 95% CI 2.54, 5.18, p < 0.0001, p for trend < 0.0001; model I, OR = 1.70, 95% CI 1.14, 2.53, p = 0.0092, p for trend = 0.0125; model II, OR = 1.88, 95% CI 1.02, 3.47, p = 0.0425, p for trend = 0.0341) compared with the lowest tertile. In model II, which was adjusted for all covariates, Participants in the highest MLR tertile had an 88% increased risk of severe AAC.

**Table 3:** Multivariate logistic regression models of severe AAC (AAC score > 6) with MLR.

	Unadjusted	Model I	Model II
	OR (95% CI) p-value	OR (95% CI) p-value	OR (95% CI) p-value
**MLR×10**	1.34 (1.24, 1.44) <0.0001	1.11(1.02, 1.22) 0.0167	1.14 (1.00, 1.31) 0.0541
**Categories**			
Tertile 1	1.0	1.0	1.0
Tertile 2	1.96 (1.34, 2.86) 0.0005	1.50 (1.00, 2.25) 0.0504	1.38 (0.74, 2.58) 0.3183
Tertile 3	3.63 (2.54, 5.18) <0.0001	1.70 (1.14, 2.53) 0.0092	1.88 (1.02, 3.47) 0.0425
p for trend	<0.0001	0.0125	0.0341

Model I adjust for age, gender, education level, and race; Model II adjusts for age, gender, education level, race, diabetes, hemoglobin A1c, BMI, AST, ALT, urea nitrogen, drinker, SBP, DBP, kidney disease, total cholesterol, smoker, uric acid, hypertension, and serum creatinine.

Subgroup analysis was performed to explore the robustness of the association between MLR and AAC score in different population setting (Table 4). The authors also conducted an interaction test to evaluate the modifier on this relationship of MLR and AAC score. Each stratification adjusted for all the factors (age, gender, education level, race, diabetes, hemoglobin A1c, BMI, AST, ALT, urea nitrogen, drinker, SBP, DBP, kidney disease, total cholesterol, smoker, uric acid, hypertension, and serum creatinine) except the stratification factor itself. There was a significant p-value for the interaction of age that affected the association between MLR and AAC score (p for interaction = 0.0346). Stronger associations between the MLR and AAC score were detected in older age (β = 0.32, 95% CI 0.14, 0.50, p = 0.0005 in participants equal to or greater than 60 years, β = 0.02, 95% CI -0.20, 0.24, p = 0.8412 in participants less than 60 years). There was a borderline significant p-value for the interaction of diabetes that affected the association between MLR and AAC score (p for interaction = 0.0578). A stronger association was detected in diabetes compared with non-diabetes (β = 0.49, 95%CI 0.16, 0.83, p = 0.0042 in participants with diabetes, β = 0.14, 95% CI -0.01, 0.29, p = 0.0617 in participants without diabetes). In addition, an interaction test in gender, BMI, hypertension, and kidney disease was conducted. However, no correlation with the p for interaction meeting the statistical significance was detected.

**Table 4:** Subgroup analysis for the association between MLR and AAC score.

	n	β (95% CI)	p-value	p for interaction
**Age**				
< 60	1626	0.02 (-0.20, 0.24)	0.8412	0.0346
≥ 60	1419	0.32 (0.14, 0.50)	0.0005	
**Gender**				
Male	1468	0.20 (0.03, 0.37)	0.0213	0.8936
Female	1577	0.18 (-0.03, 0.40)	0.0975	
**BMI**				
< 24	656	0.28 (-0.05, 0.62)	0.0999	0.7158
≥ 24, < 28	900	0.26 (0.03, 0.50)	0.0301	
≥ 28	1467	0.16 (-0.04, 0.35)	0.1120	
**Diabetes**				0.0578
Yes	506	0.49 (0.16, 0.83)	0.0042	
No	2417	0.14 (-0.01, 0.29)	0.0617	
**Hypertension**				
Yes	1441	0.16 (-0.01, 0.34)	0.0640	0.5664
No	1601	0.24 (0.03, 0.45)	0.0244	
**Kidney disease**				
Yes	117	-0.06 (-0.57, 0.44)	0.8087	0.2459
No	2924	0.24 (0.10, 0.39)	0.0010	

Data are adjusted for age, gender, education level, race, diabetes, hemoglobin A1c, BMI, AST, ALT, urea nitrogen, drinker, SBP, DBP, kidney disease, total cholesterol, smoking, uric acid, hypertension, and serum creatinine.

## Discussion

In this cross-sectional study of 3045 participants, the authors observed a positive correlation between increased monocyte lymphocyte ratio and abdominal aortic calcification in US adults. To our knowledge, the present results were the first to show that AAC scores increased as the MLR increased. The present study's findings from this study suggest that the monocyte lymphocyte ratio should be considered in patients with AAC in a clinical setting.

Multiple studies have shown a positive relationship between MLR and cardiovascular disease. In a study investigating the relationship between MLR and cardiovascular mortality, MLR was a strong and independent predictor of all-cause and cardiovascular mortality among hemodialysis patients.^20^ Wen et al. found that higher MLR levels at peritoneal dialysis initiation may be independently associated with increased CVD mortality in peritoneal dialysis patients.^21^ Reiko et al. reported that the relative risk of CVD events was 2.43 in the high MLR group compared to the low MLR group after adjusting for age, sex, and diabetes in incident dialysis patients.^22^ This is the first study showing a positive association between MLR and AAC, which is consistent with previous significant negative impacts of MLR on cardiovascular health.

Although monocyte lymphocyte ratio has been recognized as a potential risk factor for cardiovascular disease, its detailed mechanisms remain unclear. One possible mechanism is that elevated MLR may indicate an increased inflammatory response and an impaired immune response.^23^ Monocyte counts correlate with levels of pro-inflammatory mediators in the circulatory system.^24^ It is well known that the main sources of proinflammatory cytokines, such as IL-6 and TNF-α, are Peripheral Blood Mononuclear Cells (PBMCs), which consist of lymphocytes and monocytes/macrophages. The synthesis and secretion of TNF-α in monocytes, furthermore accelerate vascular calcification in uremia.^25^ Chronic low-grade inflammation, characterized by elevated concentrations of proinflammatory mediators in the circulatory system, has been identified as mediating vascular dysfunction in the general population and associated with increased CVD risk.^26^ Recent studies provided compelling evidence that vascular calcification is associated with inflammatory status and is enhanced by inflammatory cytokines.^27^, ^28^, ^29^

The subgroup analysis found that the association between MLR and AAC was more pronounced in older individuals. Most elder individuals develop inflammageing, a condition characterized by elevated blood levels of inflammatory markers that contributes significantly to chronic disease, disability, frailty, and premature death.^30^ Moreover, a stronger association was detected in diabetes compared with non-diabetes. There is an increased disease burden and higher levels of arterial calcification in diabetes patients. Advanced glycation end products treatment of Vascular Smooth Muscle Cells (VSMCs) promotes calcification through multiple mechanisms including increasing levels of alkaline phosphatase, a bone matrix protein, decreased expression of VSMCs markers, and increased expression of Runx2.^31^ These results also remind us that in elderly or diabetes patients the abnormality of MLR needs to be paid enough attention.

The present research has several limitations. Due to the single country enrolled study, the authors may not be able to reflect conclusions to a multi-ethnic or worldwide cohort. Second, based on the present results, the authors reveal that higher MLR may increase the risk of AAC, but we cannot further reveal the causal relationship of MLR to ACC. Third, the authors cannot completely exclude the effect of other possible confounding factors for these data were not available in the NHANES study design, for example, some other comorbidities including aortic aneurysm and the use of drugs.

## Conclusion

In conclusion, the present study showed that in adults over the age of 40 in the United States, elevated MLR was associated with elevated AAC scores and the risk of developing severe AAC. However, further studies are still needed to validate the present findings.

### Authors’ contributions

Peiyuan Zuo designed this study and analyzed the data. Wei Hu and Ranran Xu collected the data. Song Tong completed the organization and writing of this article. Liya Hu revised the manuscript. Both authors approved the final manuscript.

## Funding

This work was funded by grants from the National Natural Science Foundation of China (nº 81901428).

## Declaration of Competing Interest

The authors declare no conflicts of interest.
